# Combustion Synthesis of Non-Precious CuO-CeO_2_ Nanocrystalline Catalysts with Enhanced Catalytic Activity for Methane Oxidation

**DOI:** 10.3390/ma12060878

**Published:** 2019-03-15

**Authors:** Abdallah F. Zedan, Amina S. AlJaber

**Affiliations:** 1 Department of Laser Applications in Metrology, Photochemistry and Agric., National Institute of Laser Enhanced Sciences, Cairo University, Giza 12613, Egypt; 2 Department of Chemistry and Earth Sciences, Faculty of Arts and Sciences, Qatar University, Doha 2713, Qatar; a.s.aljaber@qu.edu.qa

**Keywords:** methane combustion, mixed oxides, heterogenous catalysis, solid-solution, copper-ceria, solid solution

## Abstract

In this study, xCuO-CeO_2_ mixed oxide catalysts (Cu weight ratio x = 1.5, 3, 4.5, 6 and 15 wt.%) were prepared using solution combustion synthesis (SCS) and their catalytic activities towards the methane (CH_4_) oxidation reaction were studied. The combustion synthesis of the pure CeO_2_ and the CuO-CeO_2_ solid solution catalysts was performed using copper and/or cerium nitrate salt as an oxidizer and citric acid as a fuel. A variety of standard techniques, including scanning electron microscopy (SEM), energy dispersive X-ray spectroscopy (EDX), X-ray diffraction (XRD), thermo-gravimetric analysis (TGA), X-ray photoelectron spectroscopy (XPS) and Raman spectroscopy were employed to reveal the microstructural, crystal, thermal and electronic properties that may affect the performance of CH_4_ oxidation. The CuO subphase was detected in the prepared solid solution and confirmed with XRD and Raman spectroscopy, as indicated by the XRD peaks at diffraction angles of 35.3° and 38.5° and the A_g_ Raman mode at 289 cm^−1^, which are characteristics of tenorite CuO. A profound influence of Cu content was evident, not only affecting the structural and electronic properties of the catalysts, but also the performance of catalysts in the CH_4_ oxidation. The presence of Cu in the CeO_2_ lattice obviously promoted its catalytic activity for CH_4_ catalytic oxidation. Among the prepared catalysts, the 6% CuO-CeO_2_ catalyst demonstrated the highest performance, with T_50_ = 502 °C and T_80_ = 556 °C, an activity that is associated with the availability of a fine porous structure and the enhanced surface area of this catalyst. The results demonstrate that nanocrystalline copper-ceria mixed oxide catalysts could serve as an inexpensive and active material for CH_4_ combustion.

## 1. Introduction

The catalytic oxidation of methane (CH_4_) is an important combustion reaction that has received great attention in recent years for its importance in energy and heat production [1,2,3,4,5]. Moreover, the capability to find nonconventional oxidation processes to achieve the combustion of CH_4_ into water and carbon dioxide at lower temperatures and reduce gaseous pollutants, such as carbon monoxide, is particularly important for the environment [6,7]. In addition, the release of residual CH_4_, which is commonly found in the exhaust stream of vehicles that use natural gas as fuel, imposes a significant concern to the environment since methane is a potent greenhouse gas [8]. Therefore, there is currently a pressing need for the development of catalyst systems with increased catalytic activity and lower methane conversion temperatures, integral when biogas or natural gas are combusted. Supported precious metal catalysts such as Pd and Pt exhibit high activity for CH_4_ oxidation [9,10,11,12]. However, the high cost and low thermal stability at high temperatures with the subsequent decomposition of active species make these catalysts not fully satisfactory and raises the need for alternative inexpensive catalysts [8,13]. Recently, ordered perovskite-type metal oxide nanostructures have been shown to exhibit promising thermal stability and catalytic activity toward the catalytic combustion of CH_4_, which has brought the substitutes of the precious metal catalysts into the spotlight [14,15]. Moreover, solid-solution materials based on transition metals and rare-earth oxides, such as copper and ceria, have drawn great attention as heterogenous catalysts due to their low cost and good activity for catalytic oxidation reactions [16,17]. Ceria (CeO_2_) is a rare-earth metal oxide with unique versatile properties. It has been exploited in a wide variety of applications, such as in gas sensing, solid electrolytes, oxygen membranes, environmental chemistry and heterogenous catalysis [18]. CeO_2_ exhibits significant oxygen release/storage capabilities because of the fast redox interchange of the associated Ce^4+^ and Ce^3+^ ionic species, which are related to the nature of the surface defects [19]. Nanocrystalline and porous CeO_2_ offers high tendency to the formation of large amounts of oxygen vacancies due to the feasible redox potential of Ce and the ability to maintain the electroneutrality of the Ce and O species [20,21]. The incorporation of a foreign metal such as lanthanide or transition metals onto the CeO_2_ lattice can change its chemical and electronic properties and is therefore expected to enrich the redox properties of CeO_2_ in the final metal/oxide composition, leading to higher oxygen mobility and active sites, as well as improved catalytic activity [22,23]. However, the catalytic activity of CeO_2_ is liable to be decreased, especially at elevated temperatures. This is because of the possible sintering and structural deterioration that could decrease the number of active sites [24]. On the other hand, nanocrystalline Cu-based materials have been shown to be suitable for non-precious heterogenous catalysts, with notable performance in gas-phase catalytic oxidation reactions [25,26]. The synergetic interactions between copper and cerium in copper-ceria catalytic systems could lead to improved activity for catalytic oxidation reactions [27,28]. In addition, Cu/CeO_2_ nanocatalysts have been shown to possess high activity for CO_2_ hydrogenation and the production of CO via the reverse water-gas shift (RWGS) reaction [29]. Moreover, mixed CuO-CeO_2_ oxides have been shown to exhibit high performance towards the decomposition of N_2_O, both in oxygen-rich and oxygen-poor reaction conditions [30]. In a recent study, the CuO supported on H_2_-CeO_2_ with an intrinsically high concentration of Frenkel-type oxygen vacant sites was demonstrated as a kind of promising catalyst to convert carbon dioxide into hydrogen through the water-gas shift (WGS) reaction [31]. In these studies, an improved performance is typically observed in cases of the mixed oxide catalysts compared to the corresponding individual counterparts. This is due to enhanced redox processes via synergistic interactions at the metal-support interface. Several mechanistic studies on Cu-Ce-O systems have suggested that the enhanced redox properties, including the change in the oxidation state of both copper and cerium in copper-ceria catalyst systems, can promote the rates of catalyzed reactions at the metal oxide interface [27,29,31]. Such a redox mechanism is prevailed by the formation of a Cu^1+^ species (Cu^2+^↔Cu^1+^) in CuO, which is associated with the reduction of adjacent CeO_2_ (Cu^4+^↔Cu^3+^) [32]. There are several synthesis methods which have been reported for the preparation of Cu-Ce-O catalyst systems and it has been shown that the catalyst preparation method can affect copper dispersion and interaction with ceria, which in turn can impact the redox properties of the catalyst, resulting in different catalytic performances [33,34,35,36,37,38]. In this study, nanocrystalline CuO-CeO_2_ solid mixed-oxide powders were prepared by solution combustion synthesis (SCS) as a simple and efficient method to obtain nanocrystalline and highly porous materials without the need for any treatment after synthesis. The ultrafine nature and purity of the obtained nanocrystalline porous solids can lead to enhanced rates of oxidation reactions [37]. In our synthesis, the cerium and copper nitrate salts were used as oxidizer agents and citric acid was employed as a complexation agent. We used fuel with a fuel-to-oxidizer ratio of 1.5:1 to ensure combustion under fuel-rich conditions. The Cu wt.% varied (1.5, 3, 4.5, 6 and 15%) and the catalytic activity of the pure CeO_2_ and the different nanocrystalline CuO-CeO_2_ catalysts for the methane oxidation reaction were studied. 

## 2. Materials and Methods 

### 2.1. Materials 

The chemicals used in this work were purchased from a local supplier (ITS, Doha, Qatar) and were used as-is without any treatment steps. These chemicals were as follows: Anhydrous citric acid (99.5% GPR, BDH, London, England), copper-(II) nitrate-trihydrate (98%, Purum, Sigma-Aldrich, St. Louis, MO, USA) and cerium-(III) nitrate hexahydrate (trace metal basis 99%, Sigma-Aldrich). For the preparation of all solutions, ultrapure deionized water (type 1, Direct-Q 5UV, Millipore, Molsheim, France) was used.

### 2.2. Methods

#### 2.2.1. Solution Combustion Synthesis of CeO_2_ and the CuO-CeO_2_ Catalysts 

The pure cerium oxide and different copper-cerium mixed oxide solid catalysts were prepared with the solution combustion synthesis (SCS) under fuel-rich conditions using citric acid (C_6_H_8_O_7_) as a chemical fuel and metal salts of Ce(NO_3_)_3_·6H_2_O and Cu(NO_3_)_2_·3H_2_O as oxidizers. For the synthesis of the ultrafine porous solids of CeO_2_ and CuO-CeO_2_, separate stock solutions (0.5 mol L^−1^) of citric acid, cerium nitrate and copper nitrate were first prepared by dissolving the precursors in ultrapure deionized water, followed by sonication and stirring for 10 min. The preparation of the pure CeO_2_ involved mixing appropriate volumes of cerium nitrate and citric acid stock solutions. The synthesis of CuO-CeO_2_ involved mixing predetermined volumes of copper nitrate in addition to the citric acid and cerium nitrate to achieve the desired copper weight ratio in the final mixed oxide composition. The weight ratio was between 1.5 wt.% and 15 wt.%. In preparation of all catalysts, the volume used from the 0.5 mol L^−1^ citric acid solution was determined so that the ratio of the moles of citric acid to the total moles of metal ions was 1.5:1 (mol%). The amount of citric acid was slightly in excess when compared to the total metal salts amounts. This was done to ensure the complexation of all metal content and provide fuel-rich conditions during the solution combustion process. The combustion mixture of the fuel and metal nitrate precursors was transferred to a beaker which was heated in a sand-bath under continuous stirring using a hotplate/stirrer, set at temperature of 90 °C. The mixture was kept under stirring and heating to conditions to evaporate the excess water and form a gel. The beaker containing the gel was then transferred with its content to an electrical muffle furnace which was pre-heated to a temperature of 380 °C. The beaker was kept in the muffle furnace for 4 h at this temperature until the full combustion of the gel had occurred. Upon combustion, a fluffy solid material with a sponge-like appearance was obtained. The sponge-like solid material was finally subjected to calcination for 4 h at 550 °C, with a temperature ramp rate of 5 °C min^−1^ in static air. The resulting calcined solid material was finally ground into an ultrafine powder for further use in the analysis and catalysis measurements.

#### 2.2.2. Characterization

To study the morphology and chemical composition of the as-prepared samples, scanning electron microscope (SEM) micrograph images and energy dispersive X-ray (EDX) spectra were obtained using a FEI scanning electron microscope (NOVA-NANOSEM 450, Brno, Czech Republic) equipped with an X-ray detector. X-ray diffraction (XRD) analysis was performed on a powder X-ray diffraction system (MiniFlex II, Rigaku, Tokyo, Japan) with Cu-K_α1_ radiation, operating at a power of 20 mA and 30 kV. The diffraction spectra were collected at room temperature in a 2θ diffraction angle range from 20° to 80° with a scanning rate of 0.025° step size and one step per second. The crystallinity and phase compositions of the selected powder materials were determined based on comparing the obtained diffraction patterns with those of the Joint Committee on Powder Diffraction Standards-International Center for Diffraction Data (JCPDS-ICCDD) database system. The average crystallite sizes of the selected samples were estimated from the XRD patterns based on the diffraction peak broadening, using the Scherrer equation: d=(0.94 λ)/(β·cosθ), where *d* is the average crystallite size, λ is the wavelength of the Cu-K_α1_ X-ray radiation source (1.54 nm), β is the full-width at half-maximum (FWHM), representing the broadening of the diffraction peak and θ is the angle of the X-ray diffraction [22]. The analysis was conducted based on diffraction and broadening information, using the most intense (100%) XRD reflection that was displayed at a small angle value (111). The lattice strain (%) magnitude was calculated using the X’Pert High-Score Plus software (v. 2.1.0, PANalytical, Westborough, MA, USA). Thermal gravimetric (TGA) measurements were performed on Perkin Elmer thermal gravimetric analyzer (Pyris 6, Groningen, Netherlands) in a temperature range of 50 °C to 700 °C under ambient air with a temperature ramp rate of 10 °C min^−1^. To study the surface and defect properties of the prepared samples, measurements of X-ray photoelectron spectroscopy (XPS) were performed in an ultra-vacuum chamber (approximately 5 × 10^−9^ Torr) using a Kratos Axis Ultra X-ray photoelectron spectrometer (Kratos Analytical, Manchester, UK) with a Mono Al Kα radiation source (1486.6 eV). Spectra were obtained under XPS conditions of a constant analyzer pass energy of 20.0 eV, 10 mA emission current and 15 kV anode HT. For calibration purpose, the XPS peak of C1s at 285 eV was used as a reference for all binding energies and as a correction of the surface charging effect. Raman spectra were acquired using a Thermo Scientific DXR2 spectrometer/microscope with a 50× objective (Thermo Fisher Scientific, Madison, WI, USA). The excitation was achieved using a 780 nm solid-state laser source with a laser power of 5 mW. The acquisition of the spectra involved 20 accumulations with a 4 cm^−1^ spectral resolution and a 5 min total acquisition time. 

#### 2.2.3. Methane Oxidation Catalysis Measurements

Experiments on the catalyzed oxidation of methane (CH_4_) were conducted to evaluate the performance and activity of the prepared catalysts. The experimental measurements were conducted using a customized fixed-bed continuous flow catalytic reactor connected with an online infrared gas analyzer, as described earlier [39,40,41]. The reactor was equipped with a quartz tube with a 10 mm inner diameter and the tube was heated by a split tube furnace with a multi-step temperature controller (Mini-Mite, Lindberg/Blue M Tube Furnace, Thermo Fisher Scientific). For all experimental measurements, 50 mg of the test sample was charged into the tube inside a bed of quartz wool. A stainless steel thermocouple of k-type was directly attached to the catalyst bed to measure the temperature of the catalyst. The flow mixture of the feed gas contained 1000 ppmv CH_4_ and 20% (*v*/*v*) O_2_ balanced by argon (Ar) and was passed through the catalyst bed at a total flow rate of 65 cm^3^ min^−1^ (Weight hourly space velocity (WHSV) of 78,000 cm^3^ g^−1^ h^−1^). The flow rate of the inlet gas was controlled with a HI-TEC mass flow controller (DMFC, Model: F-201CV-10K-AGD-22-V, Bronkhorst, Ruurlo, Netherlands). The outlet gas was passed into an inline infrared (IR) gas analyzer (multichannel, IR200, Yokogawa, Japan) to monitor the CH_4_ conversion by the simultaneous analysis of the composition of the flue gas. The readings of the IR gas analyzer included CH_4_, CO and CO_2_ and were simultaneously recorded and logged, along with the temperature of the catalyst during the experiment. The measurements were conducted at ambient pressure and the light-off curves were obtained by heating the reactor from ambient temperature to 600 °C at a rate of 10 °C min^−1^. The catalytic activity was expressed by the conversion of CH_4_ in the effluent gas and was indicated as CH_4_ conversion percentage (%), which was calculated as follows:

(1) $${\mathrm{CH}}_{4}\;\mathrm{Conversion}\;\left(\%\right)=\left[\frac{{\mathrm{CH}}_{4}\left(\mathrm{in}\right)-{\mathrm{CH}}_{4}\left(\mathrm{out}\right)}{{\mathrm{CH}}_{4}\left(\mathrm{in}\right)}\right]\;\times \;100,$$

For the sake of comparison, the catalyst with best performance was prepared from a different patch and its catalytic activity was tested, where it showed a similar catalytic activity. In addition, the repeatability of the experimental catalysis measurements was confirmed by conducting two separate runs for each catalyst, and the performance of the catalyst in the two subsequent tests was similar.

## 3. Results

### 3.1. Morphological and Crystal Structure of the Catalysts 

Pure and mixed rare-earth and transition metal oxides can be synthesized by several physical and wet-chemical methods, such as sonochemical [33], mechanical mixing [25], chemical precipitation [42], freeze-drying [34], conventional hydrothermal synthesis [35], microwave-assisted synthesis [38], sol-gel preparation [43] and solution combustion synthesis [36]. Solution combustion synthesis (SCS) has received a great deal of interest because of its ability to yield high-surface area materials, with ease of scalability, minimal preparation steps and almost no post-synthesis treatment is needed, significantly reducing the time needed for preparation and processing, allowing a simple and rapid obtainment of solid products [44,45,46]. The SCS is a self-sustained high-temperature thermal process involving a sol-gel medium that undergoes a self-propagating exothermic reaction between the chemical fuel and the metal oxidizer, yielding large amount of gaseous products and ultrafine solid materials [46]. In this work, we have synthesized nanocrystalline CuO-CeO_2_ solid mixed-oxide powders by SCS, using cerium and copper nitrate salts as oxidizers and citric acid as a complexation and fuel agent, with a fuel-to-oxidizer(s) ratio of 1.5:1 to ensure fuel-rich conditions. 

**Figure 1:**
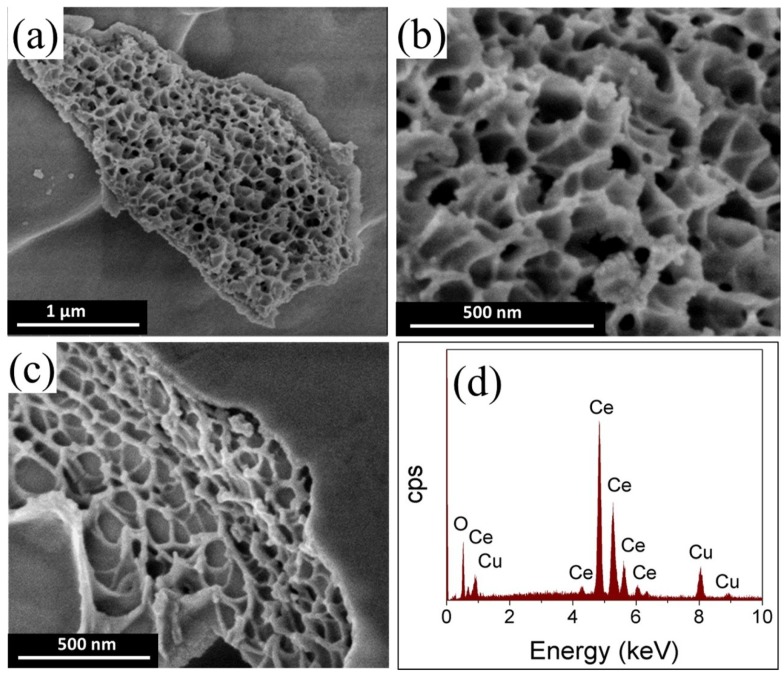
SEM micrograph images of (**a**,**b**) CeO_2_; (**c**) 6% CuO-CeO_2_ prepared by the solution combustion method, and (**d**) The EDX spectrum of 6% CuO-CeO_2_, showing the Cu and Ce elements.

Figure 1 presents the SEM micrograph images of CeO_2_ and 6% CuO-CeO_2_ (Figure 1a–c) and the EDX analysis (Figure 1d) of the 6% CuO-CeO_2_ materials synthesized by the solution combustion method. The SEM images of pure CeO_2_ shown in Figure 1a,b reveal a spongy-like morphology with a macro porous coral reef-like structure. The CuO-CeO_2_ mixed oxide with 6 wt.% copper displays a spongy-like features with large voids and small spherical agglomerates of CuO, as can be seen from SEM image shown in Figure 1c. The surface voids are formed due to the release of excessive volumes of gases upon the combustion reaction, introducing porosity to the prepared CuO-CeO_2_ materials, and leading to a reduction in the size of structural features. The doping with copper is evident from the EDX spectrum of 6% CuO-CeO_2_, shown in Figure 1d. 

**Figure 2:**
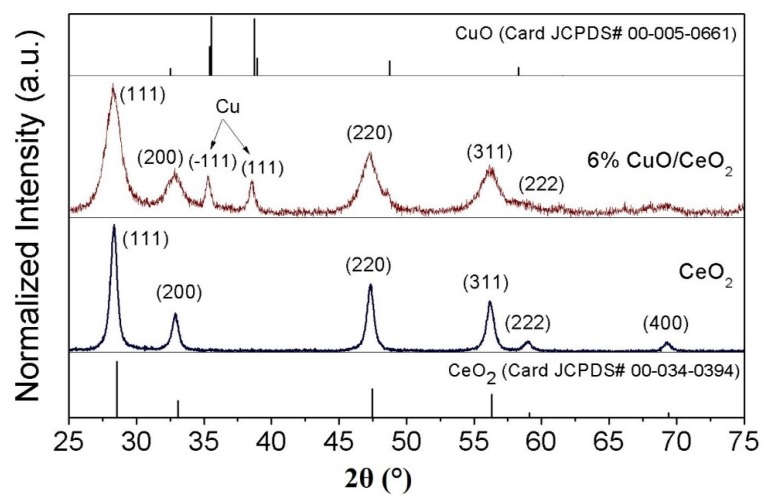
XRD patterns of the CeO_2_ and 6% CuO-CeO_2_ powders prepared using the solution combustion method compared to reference patterns of CeO_2_ and tenorite CuO.

The XRD patterns of the pure CeO_2_ and selected CuO-CeO_2_ mixed oxide with 6 wt.% copper, along with the reference patterns of CeO_2_ and tenorite CuO, are shown for the purpose of comparison in Figure 2. For the pure CeO_2_ prepared by combustion synthesis, four main XRD peaks were observed at diffraction angles of 2θ = 28.3°, 32.8°, 47.3° and 56.2° and 58.9°, corresponding to lattice planes of (111), (200), (220) (311) and (222). This is characteristic of the standard fluorite cubic lattice of ceria (Card JCPDS No. 00-034-0394). The doping of CeO_2_ with CuO resulted in the appearance of two XRD peaks at diffraction angles of 35.3° and 38.5° in the case of 6% CuO-CeO_2_, characteristic of the tenorite phase, also in accordance with the reference XRD pattern of CuO (Card JCPDS No. 00-005-0661). These two new XRD features indicate the formation of a mixed oxide solid solution. The 100% main diffraction peak at a diffraction angle of 28.3° clearly broadened when 6 wt.% copper was incorporated into the lattice of the CeO_2_, and the full width at half maximum (FWHM) increased from ~0.55° in case of CeO_2_ to ~1.1° in case of 6% CuO-CeO_2_, indicating the reduction of the size features when the copper was introduced into the ceria lattice. The crystallite average sizes of the CeO_2_ and 6% CuO-CeO_2_ materials were calculated using the Scherrer formula and it was found that the incorporation of 6 wt.% Cu led to a reduction in the mean crystallite size from ~19 nm in case of pure CeO_2_ to ~9 nm in case of CuO-CeO_2_. This reduction in size features which is associated with the main XRD peak broadening results from the refinement of the CuO-CeO_2_ mixed oxide crystallite size, due to the competitive growth between the CuO and CeO_2_ phases of the mixed oxide solid. 

These XRD results agree with the above presented SEM results (Figure 1a–c) regarding the existence of CuO as a separate phase and the decrease of the mean crystallite size resulting from the copper insertion into the CeO_2_ lattice, as indicated by larger voids in the porous structure. This decrease in size reveals the role of copper insertion in the beneficial decrease of the Cu-Ce mixed oxide crystal growth. The calculated lattice strain of the CeO_2_ and CuO-CeO_2_ further evidences the influence the incorporation of copper into the lattice of the ceria. The lattice strain value (%) increased from 0.9% in the case of CeO_2_ to 1.8% in the case of 6% CuO-CeO_2_ compared to the standard structures. This lattice strain can be ascribed to the lattice distortion and contraction resulting from the insertion of divalent Cu cations (Cu^2+^) with relatively smaller ionic radii (0.73 Å) into the ceria lattice, with Ce^4+^ cations having radii of 0.97 Å [47]. 

### 3.2. Thermal and Electronic Properties (TGA and Raman)

**Figure 3:**
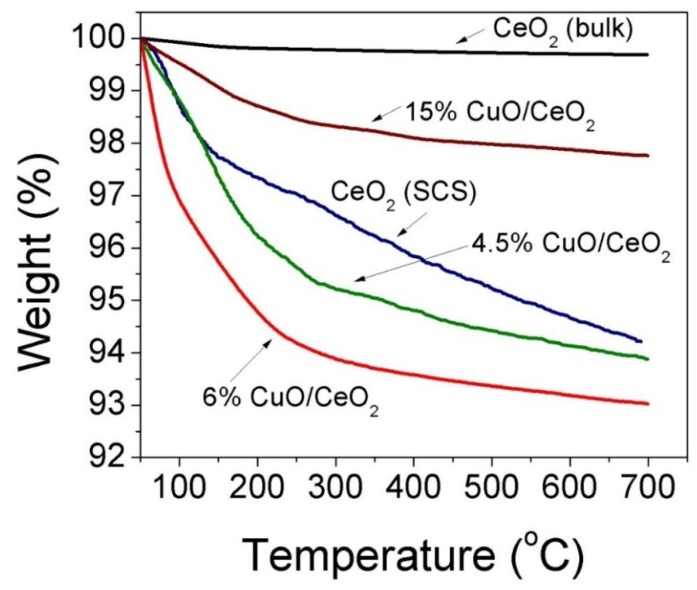
TGA profiles of (**a**) bulk ceria (**b**) CeO_2_, (**c**) 4.5% CuO-CeO_2_, (**d**) 6% CuO-CeO_2_ and (**e**) 15% CuO-CeO_2_ prepared by solution combustion method.

Figure 3 shows the thermal gravimetric analysis (TGA) plots of CeO_2_ and CuO-CeO_2_ prepared by combustion synthesis along with that of bulk CeO_2_ for the sake of comparison. Unlike the bulk CeO_2_ particles (Figure 3a), which showed negligible weight loss upon heating to 700 °C, the CeO_2_ particles prepared by combustion synthesis (Figure 3b) possessed a weight loss of ~2.3% when heated to 150 °C and ~6% weight loss after heating to 700 °C. This weight loss in the case of the combustion-synthesized CeO_2_ particles can be ascribed to the shrinkage of the space caused by the dehydration of the water molecules, which may be trapped in fine pores or adsorbed on the surface of the porous combustion-synthesized CeO_2_ particles. [48] Likewise, the CuO-CeO_2_ mixed-oxide particles, with Cu weight ratios of 4.5% and 6% (Figure 3c,d), exhibited weight loss percentages of ~5% and ~6% upon heating to 300 °C, respectively, which can be ascribed to the dehydration and desorption of the hydroxyl (–OH) groups on the surface of the particles [49]. On the other hand, the 15 wt.% CuO-CeO_2_ (Figure 3e) showed relatively higher thermal tolerance, with a weight loss of only ~2% when heated up to a temperature of 300 °C. This higher thermal stability is due to presence of a higher fraction of copper (15 wt.%) in the composite when compared to the CuO-CeO_2_ particles with 4.5–6 wt.% CuO. The relatively increased heating tolerance of the 15 wt.% CuO-CeO_2_ particles can be ascribed to the decrease of the number of hydrated hydroxyl groups (–OH) in the mixed oxide, since a Cu surface is hydrated with a fewer number of –OH groups when compared to the support oxide material [49]. The highest dehydration and hydroxyl group desorption observed for the 6 wt.% CuO-CeO_2_ at temperatures lower than 300 °C indicates the relatively higher capacity of the particles towards –OH group adsorption, which demonstrates the existence of a larger accessible surface on the porous structured catalyst. As will be discussed later, this larger surface accessibility could lead to the relatively higher catalytic activity of this catalyst towards methane combustion. In the same time, the 6% CuO-CeO_2_ demonstrated significant thermal stability, as indicated by the loss of only less than 7% of the total weight when heating to 700 °C, which reflects its ability to withstand the high-temperature-demanding conditions of thermochemical catalytic processes. 

**Figure 4:**
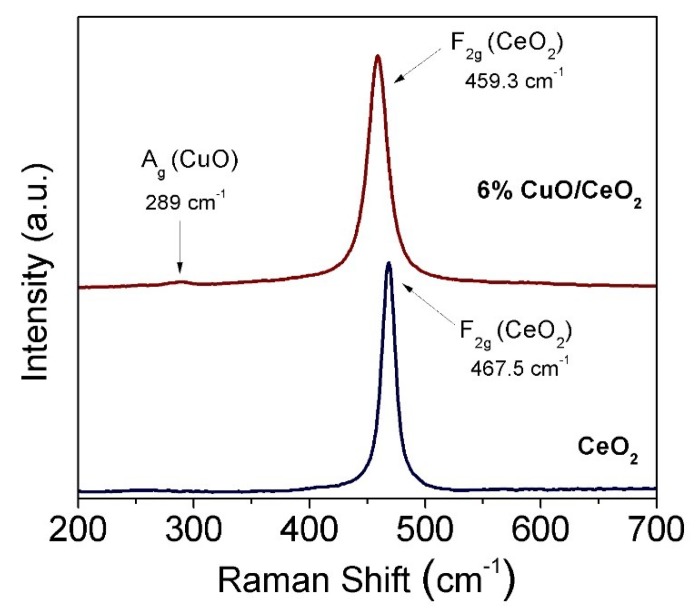
Raman spectra of CeO_2_ and 6% CuO-CeO_2_ prepared by combustion synthesis in the spectral region of 200–700 cm^−1^, showing both the F_2g_ mode of CeO_2_ and the A_g_ mode of CuO in case of the 6% CuO-CeO_2_ catalyst.

Raman spectroscopy is a powerful, sensitive, nondestructive and rapid analytical technique that can provide accurate information about the structural, symmetrical and electronic properties of nanostructures [50]. Therefore, Raman spectra of nanocrystalline pure ceria and selected copper-ceria mixed oxide solids were obtained to study the effect of the incorporation of copper on the lattice vibration features of ceria. Figure 4 displays the Raman spectra of pure nanocrystalline CeO_2_ and 6 wt.% CuO-CeO_2_ solid powders prepared by SCS. The Raman spectra of pure nanocrystalline CeO_2_ exhibits a pronounced peak centered at 467.5 cm^−1^, which is assigned to the F_2g_ lattice vibration mode, characteristic of fluorite cubic-structured ceria. The F_2g_ mode of CeO_2_ is associated with the symmetrical stretching vibrational mode of oxygen atoms around oxygen atoms in the fluorite ceria lattice [51]. The Raman spectrum of the nanocrystalline 6% CuO-CeO_2_ solid powder exhibits a main band centered at 459.3 cm^−1^ due to the F_2g_ vibration mode of the CeO_2_ lattice, in addition to a small shoulder peak at ~289 cm^−1^, characteristic of the A_g_ mode of the tenorite CuO sublattice [52]. The intercomparing of the position of the two F_2g_ bands of CeO_2_ and CuO-CeO_2_ indicates that the incorporation of copper onto ceria led to the shift of the F_2g_ band to a lower wavenumber value (red-shift) from 467.5 cm^−1^ to 459.3 cm^−1^. This red-shift can be ascribed to lattice distortion, resulting from the insertion of divalent copper cations with relatively smaller ionic radii and lower oxidation states compared to tetravalent cerium cations. This is associated with the generation of oxygen vacancies (defects) within the mixed-oxide lattice, which can lead to shortening of the cerium-oxygen bonds, resulting in an overall lattice contraction [43]. Moreover, the formation of oxygen vacancies upon the addition of copper to ceria can lead to the generation of partially reduced cerium cations in the form of trivalent ions (Ce^3+^) which can promote the catalytic activity in an oxidation reaction by enhanced oxygen diffusion [20,23]. In addition, the line-shape in the case of CuO-CeO_2_ became slightly asymmetric and broadened when compared to the pure CeO_2_, which can be attributed to the inhomogeneous strain broadening introduced by the phonon confinement and dispersion introduced as the grain size decreases upon Cu incorporation [50]. The Raman results agree with the previously discussed XRD results (Figure 2) confirming the unit cell contraction, as indicated by the shift of the X-ray diffraction and Raman peaks upon the insertion of copper. 

### 3.3. Surface Chemical Analysis (XPS) 

**Figure 5:**
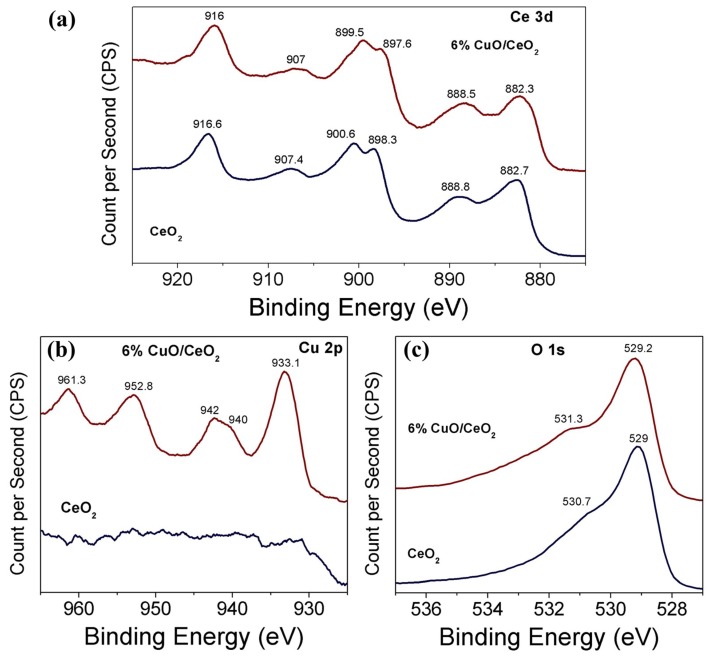
XPS high-resolution spectra of (**a**) Ce 3d, (**b**) Cu 2p and (**c**) O 1s of CeO_2_ and 6% CuO-CeO_2_ catalysts, synthesized using the solution combustion method.

XPS spectra of the selected catalysts were obtained to study the chemical and electronic speciation environment of cerium, copper and oxygen in the studied material, based on information derived from the values of the binding energies [53]. Figure 5 displays the XPS high-resolution spectra of Ce 3d, Cu 2p and O 1s of both CeO_2_ and 6% CuO-CeO_2_ catalysts synthesized using the solution combustion method. The XPS high-resolution scans were acquired between 875–925 eV, 925–965 eV and 527–537 eV for Ce 3d, Cu 2p and O 1s, receptively. The core level spectra of Ce 3d, of both CeO_2_ and 6% CuO-CeO_2_ (Figure 5a), exhibits six pronounced peaks and fewer less intense peaks. The six strong peak components are divided into two sets: A set of three peaks of Ce 3d_5/2_ level with a v structure ($v,v^{\prime \prime },v^{\prime \prime \prime }$) and another set of three peaks of Ce 3d_3/2_ level with a u structure ($u,u^{\prime \prime },u^{\prime \prime \prime }$) [54]. These pronounced six components are attributed to Ce^4+^ ions of ceria, which agrees with the results of the XRD and Raman spectroscopy discussed earlier. The other less-pronounced components are ascribed to the minor Ce^3+^ species present in ceria. The binding energies of the six Ce^4+^ components ($v,v^{\prime \prime },v^{\prime \prime \prime },u,u^{\prime \prime },u^{\prime \prime \prime }$) are 882.3, 888.5, 897.6, 899.5, 907 and 916 eV for CeO_2_ and 882.7, 888.8, 898.3, 900.6, 907.4 and 916.6 eV in case of 6% CuO-CeO_2_, respectively. The binding energy values of the six components are shifted to higher binding energies in case of a CuO-CeO_2_ mixed oxide when compared to pure CeO_2_, indicating that the chemical speciation of ceria is influenced by the interaction between the CuO and CeO_2_ in the mixed oxide lattice [55]. The Cu 2p core level spectrum of CuO-CeO_2_ (Figure 5b) is featured with noticeable peaks centered around 933.1 eV and 952.8 eV, corresponding to Cu 2p_3/2_ and Cu 2p_1/2_, respectively and shake-up satellite peaks at around 940 and 942 eV due to the presence of a Cu^2+^ species [56]. The main Cu 2p_3/2_ peak, together with the appearance of shake-up satellite peaks, constitutes the main XPS features, characteristic of a CuO sublattice in the CuO-CeO_2_ catalyst [30]. This was also confirmed in the present study by comparing the XRD analysis of the different samples. The O 1s core spectra of CeO_2_ and 6% CuO-CeO_2_ (Figure 5c) exhibit two peaks, a broad intense peak at 529 eV and a less-intense peak at 530.7 eV in the case of CeO_2_ and a broad intense peak at 529.2 eV and a less-intense peak at 531.3 in the case of CuO-CeO_2_. The main intense XPS peaks at a lower binding energy (529–529.2 eV) are ascribed to the lattice O^2−^ ions, whereas the less-intense shoulder peaks at a higher binding energy (530.7–531.3 eV) can be attributed to the hydroxyl (–OH) or polarized oxygen species present close to the oxygen vacancies [57,58]. The center of the main and shoulder peaks in the case of CuO-CeO_2_ are slightly shifted to higher binding energy (blue-shift) when compared to pure CeO_2_, indicating the influence of the difference in electronegativity between copper and cerium on the chemical speciation of oxygen species. 

### 3.4. Catalytic CH_4_ Oxidation Study

**Figure 6:**
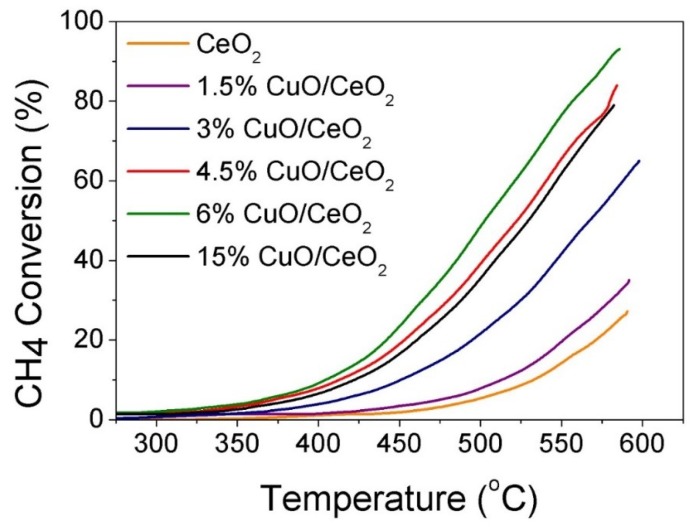
Light-off curves of CH_4_ oxidation measured under 78,000 cm^3^ g^−1^ h^−1^ WHSV for CeO_2_ and different CuO-CeO_2_ catalysts prepared by solution combustion synthesis (SCS).

**Figure 7:**
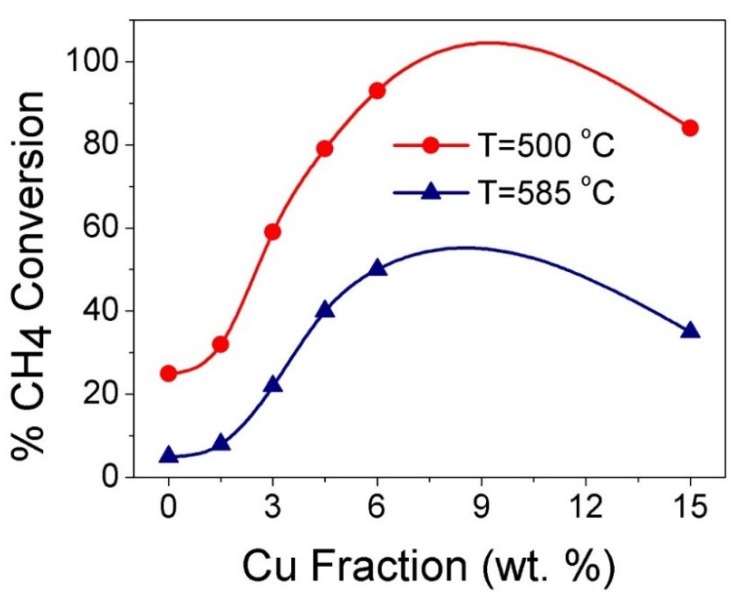
Effect of Cu content (wt.%) on the CH_4_ conversion (%) at T = 500 °C and 585 °C for different xCuO-CeO_2_ catalysts (x = 0, 1.5, 3, 4.5, 6, 15%) prepared by SCS.

The oxidation of CH_4_ over CeO_2_ and CuO-CeO_2_ prepared by SCS was studied and the measured light-off curves of CH_4_ conversion under 78,000 cm^3^ g^−1^ h^−1^ WHSV over different catalysts are presented in Figure 6. In all cases, the variation of the catalytic activity towards methane combustion was expressed as CH_4_ conversion (%) as a function of the catalyst temperature (T). The obtained results indicate that nanocrystalline mixed oxide catalysts (CuO-CeO_2_) are more active than pure CeO_2_ catalysts prepared using the same SCS method. For all of the studied catalysts, carbon dioxide was the only gaseous reaction product that was detected and no carbon monoxide gas could be detected. The methane to carbon dioxide conversion could be observed upon reaching a temperature of 350 °C (Figure 6). Upon increasing the reactor temperature above 450 °C, CH_4_ conversion significantly increases due to the increased catalytic activity of the catalysts at higher temperatures. The 6% CuO-CeO_2_ demonstrated the best catalytic activity for CH_4_ oxidation in all series, with the highest CH_4_ conversion of 93% at 585 °C, followed by the 4.5% CuO-CeO_2_ which possessed a maximum CH_4_ conversion of 84% at the same temperature of 585 °C (Table 1). Comparing the values of T_80_ (T_80_ is the temperature corresponding to 80% conversion of CH_4_) for these best two catalysts confirms the superior performance of the 6% CuO-CeO_2_ with T_80_ of 556 °C compared to 580 °C of that of the 4.5% CuO-CeO_2_ (Table 1). The catalytic activity determined from the % conversion of CH_4_ at 500 °C and 585 °C of the different tested CuO-CeO_2_ catalysts with Cu content from 1.5–15 wt.% follows the following order: 6% CuO-CeO_2_ > 4.5% CuO-CeO_2_ > 15% CuO-CeO_2_ > 3% CuO-CeO_2_ > 1.5% CuO-CeO_2_ (Table 1 and Figure 7). This order of catalytic activity is also obvious when comparing the values of T_25_ and T_50_ for the same catalysts (T_25_ and T_50_ are the temperatures corresponding to the 25% or 50% conversion of CH_4_, respectively), as can be seen in Figure 6 and Table 1. In general, all CuO-CeO_2_ mixed-oxide catalysts possessed clearly better activity when compared to pure CeO_2_, where all catalysts exhibited a T_25_ less than 565 °C (Table 1). The performance of the 15% CuO-CeO_2_ catalyst indicates that when Cu content was increased from 6 wt.% to 15 wt.%, a suppression of activity is observed, giving rise to T_25_ and T_50_ of 475 °C and 530 °C for the 15% CuO-CeO_2_ compared to 453 °C and 502 °C for the 6% CuO-CeO_2_, respectively. The lower activity of the 15% CuO-CeO_2_, with the largest Cu content, could result from the existence of a separate phase of CuO with a lower number of coordination sites relative to the ceria support, as confirmed by the TGA results discussed earlier (Figure 3). These results reveal the beneficial influence of the solid mixed-oxide solution for CH_4_ oxidation and the key role of copper oxide in activating CH_4_ combustion. The catalyst exhibiting the best performance in this study showed better activity than the ceria-supported plasmonic metal catalyst reported in previous literature. For example, the T_80_ observed for the most active 6% CuO-CeO_2_ catalyst at 556 °C in this work is significantly lower than the T_80_ value reported in the literature for the 5% Pd-CeO_2_/Al_2_O_3_ catalyst prepared by the wet impregnation method, where T_80_ was ≥700 °C [59]. In addition, the T_50_ of our 6% CuO-CeO_2_ catalyst (502 °C) is similar to that reported in the literature for a PdPt/SiO_2_ catalyst, providing the lower cost of Cu compared to PdPt metals [5]. Based on the catalytic activity results, it can be concluded that the presence of Cu^2+^ ions (hence a CuO sublattice) inserted in the ceria lattice and the consequent interaction between the two Cu and Ce oxides are keys to activating CH_4_ and improving its catalyzed oxidation rate. It is generally accepted that the catalyzed oxidation of fuels over metal oxides catalysts, including the CH_4_ oxidation reaction, follows the prevalent Mars-van Krevelen mechanism [4,60]. The CH_4_ oxidation process involves the adsorption of CH_4_ on the metal oxide surface, followed by activation of the C–H bond on an oxygen-deficient vacant site on the active catalyst surface [7]. Following the dissociative adsorption of methane and C–H bond breaking, the oxide support incorporates oxygen into the activated CH_4_ molecule from its lattice oxygen atoms which act as reactants and become part of the oxidized product molecule. The catalytic cycle is then completed through the replenishing of O_2_-deficient vacant sites through the adsorption and dissociation of O_2_ in the feed gas [3]. It can be concluded that the activity of a catalyst for CH_4_ oxidation is affected by the potency of abstracting active oxygen species and the replenishment of the O_2_-deficient vacant sites, and the ultrafine and porous nature of the 6% CuO-CeO_2_ catalyst, improves oxygen diffusion and hence the CH_4_ oxidation reaction. These results demonstrate the promising practical applicability of the non-precious CuO-CeO_2_ solid solution as an active catalyst material which can serve as a basis for the development of an effective and cheap technology for the oxidative abatement of CH_4_ emissions. 

## 4. Conclusions

In this study, a series of CuO-CeO_2_ solid solution catalysts was prepared using the solution combustion synthesis method with Cu inserted into CeO_2_ lattice. The CuO-CeO_2_ catalyst exhibited a fine porous microstructure with a coral reef-like morphology and extended voids because of the release of a large volume of gases upon the combustion of the precursor compounds. Raman spectroscopy and XRD results confirmed the existence of the CuO subphase in the prepared CuO-CeO_2_ mixed oxides. The insertion of Cu in CeO_2_ could promote the catalytic activity of the mixed oxide for CH_4_ oxidation. Among the prepared catalysts, the 6% CuO-CeO_2_ catalyst exhibited the best catalytic activity for CH_4_ oxidation because of its ultrafine and porous nature, which could facilitate lattice oxygen diffusion and hence CH_4_ combustion. The obtained results demonstrate the promise of the simple CuO-CeO_2_ mixed oxide as an active composite for practical application in methane combustion. In future work, the catalytic activity of these CuO-CeO_2_ solid solution catalysts should be further enhanced through interfacing with a proper active metal by the formation of a nanoalloy of two or three metals, with ceria, to further decrease the temperature required for the conversion of methane. This can pave the way for the practical implementation of cheap and active catalyst materials in low-cost and effective technologies for the abatement of methane emissions.

## Figures and Tables

**Table 1:** List of different catalysts prepared by SCS and their corresponding T_25_, T_50_, T_80_ and % CH_4_ conversion at two different temperatures of 500 °C and 585 °C.

Catalyst	T_25_ (°C)	T_50_ (°C)	T_80_ (°C)	% CH_4_ Conversion (T = 500 °C)	% CH_4_ Conversion (T = 585 °C)
CeO_2_	585	-	-	5	25
1.5% CuO-CeO_2_	565	-	-	8	32
3% CuO-CeO_2_	510	567	-	22	59
4.5% CuO-CeO_2_	465	522	580	40	84
6% CuO-CeO_2_	453	502	556	50	93
15% CuO-CeO_2_	475	530	-	35	79
